# Longitudinal transcriptomic characterization of viral genes in HSV-1 infected tree shrew trigeminal ganglia

**DOI:** 10.1186/s12985-020-01344-8

**Published:** 2020-07-08

**Authors:** Erlin Wang, Yunshuang Ye, Ke Zhang, Jinlong Yang, Daohua Gong, Jianhua Zhang, Renjun Hong, Huan Zhang, Lihong Li, Guijun Chen, Liping Yang, Jianmei Liu, Hanyu Cao, Ting Du, Nigel W. Fraser, Le Cheng, Xia Cao, Jumin Zhou

**Affiliations:** 1 Key Laboratory of Animal Models and Human Disease Mechanism of the Chinese Academy of Science/Key Laboratory of Healthy Aging Research of Yunnan Province, Kunming Institute of Zoology, the Chinese Academy of Sciences, Kunming, 650223 Yunnan China; 2 Key Laboratory of Second Affiliated Hospital of Kunming Medical University, Kunming, 650000 Yunnan China; 3 BGI-Yunnan, BGI-Shenzhen, Kunming, 650000 Yunnan China; 4 Kunming College of Life Science, University of Chinese Academy of Sciences, Beijing, 100049 China; 5 Shanghai Key Laboratory of Forensic Medicine, Shanghai Forensic Service Platform, Academy of Forensic Science, Shanghai, 200063 China; 6 School of Forensic Medicine, Kunming Medical University, Kunming, 650101 Yunnan China; 7 Department of Microbiology, Perelman School of Medicine, University of Pennsylvania, Philadelphia, PA19104 USA; 8 Department of medicine laboratory, Fuwai Central China Cardiovascular Hospital, Zhengzhou, 450003 Henan China; 9 College of Forensic Science, Xi’an Jiaotong University, Xi’an, 710049 Shaanxi China

**Keywords:** HSV-1, Tree shrew, RNAseq, Trigeminal ganglia, Latency, Spontaneous reactivation, Transcriptome, Longitudinal study

## Abstract

**Background:**

Following acute infection, Herpes Simplex virus-1 (HSV-1) establishes lifelong latency and recurrent reactivation in the sensory neurons of trigeminal ganglia (TG). Infected tree shrew differs from mouse and show characteristics similar to human infection. A detailed transcriptomic analysis of the tree shrew model could provide mechanistic insights into HSV-1 infection in humans.

**Methods:**

We sequenced the transcriptome of infected TGs from tree shrews and mice, and 4 human donors, then examined viral genes expression up to 58 days in infected TGs from mouse and tree shrew, and compare the latency data with that in human TGs.

**Results:**

Here, we found that all HSV-1 genes could be detected in mouse TGs during acute infection, but 22 viral genes necessary for viral transcription, replication and viral maturation were not expressed in tree shrew TGs during this stage. Importantly, during latency, we found that LAT could be detected both in mouse and tree shrew, but the latter also has an ICP0 transcript signal absent in mouse but present in human samples. Importantly, we observed that infected human and tree shrew TGs have a more similar LAT region transcription peak. More importantly, we observed that HSV-1 spontaneously reactivates from latently infected tree shrews with relatively high efficiency.

**Conclusions:**

These results represent the first longitudinal transcriptomic characterization of HSV-1 infection in during acute, latency and recurrent phases, and revealed that tree shrew infection has important similar features with human infection.

## Introduction

HSV-1 is a ubiquitous but important human pathogen carried by over half of the world’s population; HSV-1 infection starts with primary infection at the periphery and subsequent lifelong latency in the peripheral nervous system [1]. In experimental animals such as mouse, acute infection develops following cornea inoculation, the virus replicates in the epithelial cells on the corneal surface and is later transported into trigeminal ganglia, where it establishes latency [2–4]. The acute stage of infection involves lytic, or productive infection of HSV-1 at the site of inoculation, and frequently in the TGs of infected mice; in the tree shrew TGs, however, signs of lytic infected were not seen and no infectious virus could be detected [5]. During the lytic phase of infection in cultured cells, all viral genes are believed to be expressed in a cascade-dependent manner [6, 7], but during latency, most viral genes are silenced with the exception of the latency-associated transcript (LAT), multiple miRNAs [8–10] and two small RNAs [11]. Although latent, the virus reactivates from individual neurons periodically, and could cause more serious diseases including herpes keratitis or herpes encephalitis [12, 13]. Recently, HSV-1 has been suggested to play a role in Alzheimer’s disease [14, 15].

Multiple animal species including mice, rabbits and tree shrews [5, 16, 17] have been used to model human HSV-1 infection, with mice being the most widely used. However, we have recently studied HSV-1 infection in tree shrews, which are more closely related to primates than rodents [18], and observed differences between tree shrews and mice during acute infection, latency and reactivation [5, 19]. Here, we performed an in-depth transcriptional profiling of the infected mouse, tree shrew and human TGs, and reveal the differences in viral gene expression patterns. Importantly, we show that latent tree shrew TGs can express many viral genes, including UL6, UL8 and ICP8, which are consistent with spontaneous reactivation. More importantly, during latency tree shrew and human TGs had more similar transcription peaks in the LAT region and possibly more ICP0 transcription, supporting that the tree shrew model better mimics HSV-1 latent infection in human.

## Results

### Viral infection dynamics vary among animal models

**Fig. 1:**
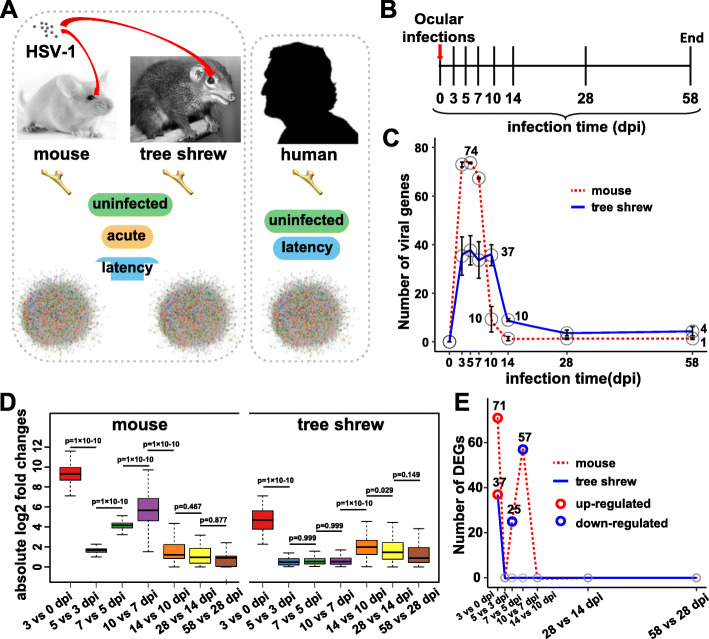
Overview of the experimental procedure. a Overview of TG collection and RNA-sequencing data used in this study. Mouse and Tree Shrew infected models were established through the ocular infection route, following which TGs were collected at different infection times for RNA sequencing. In addition to 16 published human TG data sets, we generated RNA-sequencing data sets from 4 human TGs. These RNA-seq data were used for viral transcriptome analysis in this study. Sample types were labeled in each host model: uninfected samples, acute infected samples and latent infected samples. **b** Experimental timeline for infection of animals and the subsequent collection of samples. **c** Line chart representing the total number of viral genes (read count > 10) at the indicated time points. Data are represented as mean ± standard deviation (SD), and means were labeled around points. **d** Distribution of absolute log_2_ fold changes for 74 viral genes between adjacent time points in two animal models. Lines represent medians, boxes represent 25–75% intervals, and whiskers represent 5–95% intervals. Outliers are not shown. Differences in fold changes were tested by ANOVA. **e** The lines report number of DEGs (log_2_ fold change > 1 & adjusted P value < 0.01) for each pair of consecutive time points in two animal modes

To analyze the viral transcriptional patterns during acute and latent stages of HSV-1 infection, mice and tree shrews were infected with HSV-1 strain 17+ by corneal scarification, and infected TGs collected over a period of 58 days (Fig. 1a, b). For each time point, three biological replicates were generated. Human TG samples were also collected for comparison with latently infected mouse and tree shrew TGs (Fig. 1a).

**Fig. 2:**
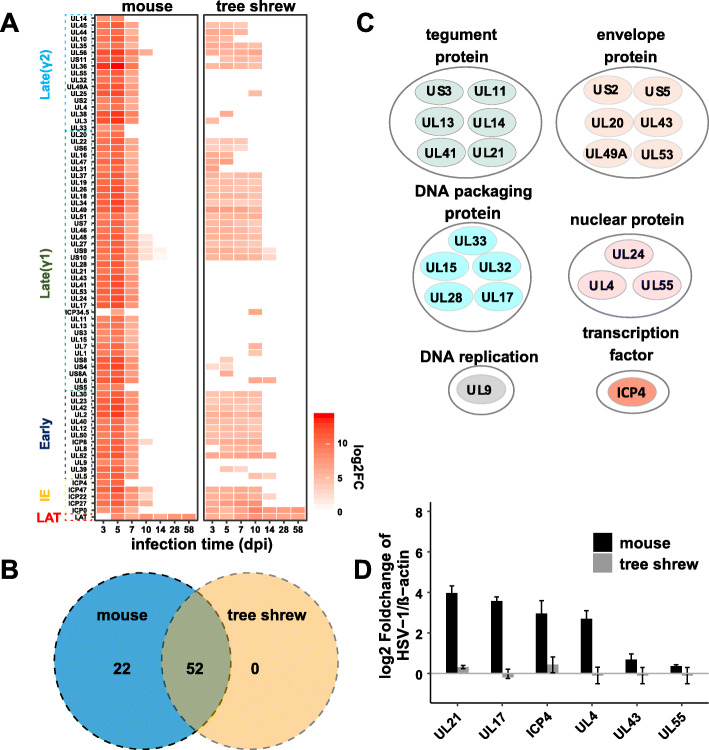
Comparison of viral gene transcription activity between mouse and tree shrew. **a** Heatmaps reflecting expression intensity of viral genes which were differentially expressed between infected samples and mock controls. Genes with a log_2_ fold change > 1 and adjusted P value < 0.01 were considered differentially expressed and were displayed in the heat map by red color. White areas indicate viral genes that below the DEGs threshold. The viral genes of different phases are distinguished by boxes and different colors on the Y-axis. IE: immediate-early. log_2_FC: log_2_ fold change. **b** Venn diagram indicating shared viral DEGs between infected mouse and tree shrew samples. **c** Functional enrichment analysis for 22 selected viral genes which are below the DEGs threshold in tree shrews. The genes annotation information used in this analysis are shown in supplemental Table 1. **d** Relative quantitation of selected viral transcripts based on the RT-qPCR validation using 5dpi RNA samples. Data are represented as mean ± SD

In total, we sequenced 52 samples (24 mouse TGs, 24 tree shrew TGs and 4 human TGs) using an Illumina HiSeq Platform and generated about 5.9 million reads per sample. After mapping to the genome of the hosts and HSV-1 strain 17+, we performed differentially expressed gene (DEG) detection using DESeq2 (differential expression analysis for sequence count data2) [20], in which we compared viral and host gene expression between samples with different infection times and uninfected control samples or each pair of consecutive time points to determine what viral or host genes are expressed significantly. The host genes were then filtered out and are not discussed further here. When using read count > 10 as the detection threshold of viral genes, we found that the number of viral genes were quite different between mice and tree shrews (Fig. 1c). Almost all of the 74 unique viral genes (LAT, ICP0, ICP34.5 and ICP4 all have a duplicated in HSV-1 genome) were expressed in mouse samples from 3 to 7 days post infection (dpi), while only a total of 52 viral genes were detected during the acute phase (3 to 10 dpi) in tree shrew TGs. The differences extended to latency, only LAT was detected in mouse TGs. However, infected tree shrew TGs also showed a high level of ICP0 transcript signal at both 28 and 58 dpi (Fig. 2a).

To provide a dynamic change in viral genes throughout the infection period, we plotted the distribution of absolute fold changes in viral genes expression for each model separately. For both models, the largest differences were observed between the 0 and 3dpi. After 3dpi, the tree shrew model showed an abrupt decline until 10dpi, then increased between 10 and 14dpi, and then slowly declined again until 58dpi. The differences were that mouse model displayed an ascending change during 5 to 10 dpi and showed no significant change in the range of 14 to 28dpi (p > 0.05, ANOVA test, Fig. 1d). Next, we asked whether there were differences in the up- or down-regulation of viral genes during this period between the two models. The number of differentially expressed genes for each pairwise comparison and each model separately was shown in Fig. 1e, which was highest in mouse model between 0 and 3dpi (up-regulated), and showed down-regulated genes within 5 to 10dpi. This demonstrates that the infected moue TG showed the largest changes in viral genes expression levels during acute phase, while in tree shrew the changes are mild in acute stage, but more significant changes occur during latency. Therefore, it appears that strong acute infections tend to lead to a calm latency, while the milder acute stage results in a latency with greater change in viral transcription and a higher frequency of reactivation.

The acute infection in mouse TG is more consistent with productive infection based on the expression of essentially all HSV-1 genes, therefore we compared mouse TG viral gene expression with that in human primary fibroblasts at 2, 4 and 8 h post infection (hpi), and found that the transcriptional pattern of lytic infection was similar to that in the mouse model (Supplemental Figure 1). When considering that mice have a higher mortality than tree shrews during the acute stage [19] and immunohistochemistry failed to detect any HSV-1 antigens in acutely infected tree shrew TGs while positive signals were detected in mouse TGs [5], these results support the conclusion that HSV-1 can build a highly active productive infection in mouse, but only mount a limited or abortive infection in tree shrew TGs.

### Viral gene expression patterns differ between infected mouse and tree shrew TGs

To determine the temporal pattern of viral gene expression following inoculation, we generated a heat map of the viral DEGs expression data. As shown in Fig. 2a, in mouse TGs essentially all HSV-1 genes were detected with higher transcription levels during the acute stage, but in tree shrew TGs, only some of these genes were detected. During latency, from day 28–58, tree shrew and mouse differed in that tree shrew TGs expressed ICP0 transcript, in addition to LAT, while mouse TGs express only LAT with little or no ICP0 signal.

Next, we focused on the viral genes that are not expressed or are expressed at extremely low levels in acutely infected tree shrew TGs, but are expressed in acutely infected mouse TGs. We found that there are at least 22 such genes (Fig. 2b). Using UniProt database and HSV-1 17+ genome annotation information in NCBI (Supplemental Table 1), we sorted these 22 viral genes into 6 clusters (Fig. 2c). Four of these clusters contain at least 3 viral genes, and these are tegument proteins, envelope glycoproteins, DNA packaging proteins and nuclear proteins, while the other clusters included ICP4 and UL9. Selected RT-qPCR validation of these transcripts is shown in Fig. 2d.

Importantly, of the 22 undetected genes, 6 are envelope proteins, functioning in viral entry and membrane fusion. Thus HSV-1 infected cells from tree shrew TGs are likely missing these essential gene products for viral maturation and subsequent infection. With most of the important late structural genes not being made, HSV-1 in acutely infected tree shrew TGs was likely undergoing an abortive lytic infection, which explains the low toxicity and absence of infectious HSV-1 in tree shrews. Furthermore, two of the viral genes not detectable in infected tree shrew TGs are ICP4 and UL9. ICP4 mainly acts as a transactivator of viral transcription, which is essential for expression of almost all early and late genes; UL9 encodes the viral origin binding protein, which acts as an ATPase and helicase and is required for initiation of DNA synthesis [1]. The lack of detectable expression of these genes could account for the fact that viral transcription is limited in tree shrew TGs.

### Many HSV-1 genes that are below the detection threshold by immunohistochemistry or immunofluorescence were expressed at extremely low levels in tree shrew TGs

**Fig. 3:**
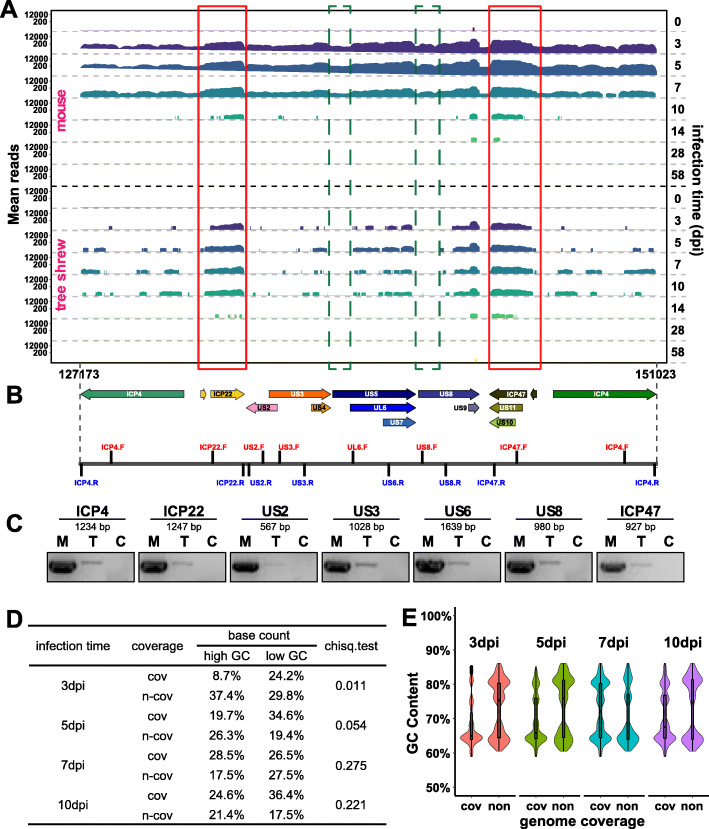
Characterization of ICP4 and other U_S_ transcripts. **a** Coverage profiles for ICP4 and serial U_S_ genes at indicated days post-infection are shown. Bedtools (v2.27.1) software and ‘genomecov’ arithmetic was used to compute the depth over the entire HSV-1 genome at each genome position. Respective transcription coordinates and chromosome regions are indicated at the bottom and infection time (dpi) are shown in the right. Data represents the average count from three biological replicates. Red box: both covered region in acute infected mouse and tree shrew samples; green box: uncovered region in acute infected tree shrew samples but in mouse samples. **b** Schematic view of PCR region of used primers. **c** PCR results of ICP4 and U_S_ genes of 5dpi TGs. M: mouse, T: tree shrew, C: blank control (ddH_2_0 template). **d** Analysis of the effect of GC content on reads coverage in ICP4 and U_S_ genes region. Chose the range from 127,173 to 151,023 bp as the analysis region. Mean reads count <= 10 were seen as “non-coverage”, and mean reads count > 10 were considered as “coverage”. Select 1 kb as a window to calculate the GC content at each site, and GC contents > = 70% were regarded as high GC loci, and < 70% were regarded as low GC loci. The coverage and number of GC sites in each acutely infected tree shrew samples were counted, and the relationship of GC content and coverage was analyzed by Chi-square test using R software. Significant: chiseq.test <= 0.05; non-significant: chiseq.test > 0.05. **e** Violin plot showing the distribution of GC content and reads coverage statue in ICP4 and U_S_ genes region. Infected time (day): 3dpi, 5dpi, 7dpi and 10dpi; coverage statue: cov = coverage, non = non-coverage

In our previous study, we could detect HSV-1 antigens by immunohistochemistry and in particular ICP4 by immunofluorescence in acutely infected mouse TGs, but we could not detect any HSV-1 proteins in acutely infected tree shrew TGs by immunohistochemistry using the same anti-HSV-1 polyclonal antibodies, or by immunofluorescence using monoclonal antibody against viral essential genes ICP0 and ICP4 [5]. Consistent with this data, ICP4 transcript was noticeably nearly absent by Real Time PCR analysis (Fig. 2d). When examining the sequencing data, we noted that ICP4 reads were extremely low. We selected ICP4 and unique short region (US) genes for further analyses, as this region also includes genes US2, US3 and US5, which are also below the detection threshold (log2 fold change > 1, and adjusted P value < 0.01). The sequence coverage profiles are shown in Fig. 3a. The maximal mean ICP4 reads in mouse TGs were very high, at nearly 12,000, contrasting with tree shrew which were only 200. A more common phenomenon in the data from acutely infected tree shrew TGs is that many viral genes show incomplete coverage in coding regions. To determine whether this incomplete coverage reflects the difference in length of the transcript, we designed primers to amplify about 1 kb fragments of selected viral genes (Fig. 3b and c). We also sequenced these PCR products and found that they correctly aligned to the viral genome. These results support the existence of very low levels of intact transcripts in areas with little or no reads in RNAseq.

The uneven coverage is seen in many HSV-1 genes and is likely due to the low efficiency in amplification during sequencing as a result of the high GC-content of the viral genome [21]. Consistent with this possibility, the detected reads correspond to reads that are also abundant in mouse samples (see red boxes in Fig. 3a), while the region with little or no reads correspond to relatively low reads in the mouse samples (see green boxes in Fig. 3a). We assessed the relationship between GC-content and genome coverage in tree shrew acute stage samples (Fig. 3d and Supplemental Figure 2A). These result support that the “coverage” or “non-coverage” in viral genome is not related with GC-content in the case of a large amount of virus genes transcription (tree shrew 5dpi, 7dpi and 10dpi samples); however, if HSV-1 genome has a low transcriptional activity, GC-content has a significant effect on genome coverage. The non-covered region in tree shrew samples both has low GC-content sites and high GC-content sites (Fig. 3e and Supplemental Figure 2B), which indicated that high GC-content is not the only reason for those undetectable viral genes in tree shrew samples.

Since ICP4 is essential for viral transcription, it’s extremely low level of expression contributes to the lack of a productive infection in tree shrew TGs [22–25]. The lack of ICP4 is likely due to transcriptional inhibition and not a result of degradation of the ICP4 protein. In our previous study, we had reported that HSV-1 could become latent and later spontaneously reactivate from tree shrew TGs, and that the virus could mount a full productive infection in tree shrew CNS in vivo [19], and in vitro in cultured ganglion neurons from tree shrew TGs (Supplemental Figure 3), thus, ICP4 protein can function in tree shrew neurons, and the low expression is more likely due to the specific inhibition of the ICP4 transcription in TG neurons in vivo. Nonetheless, we believe, that the extremely low level of ICP4 transcripts might have been sufficient for the virus to sustain a low level of genome replication as reported before [26] and subsequently achieve latency in the tree shrew without triggering a full blown infection and immune response.

### Spontaneous reactivation is detected during latency in tree shrew TGs

**Fig. 4:**
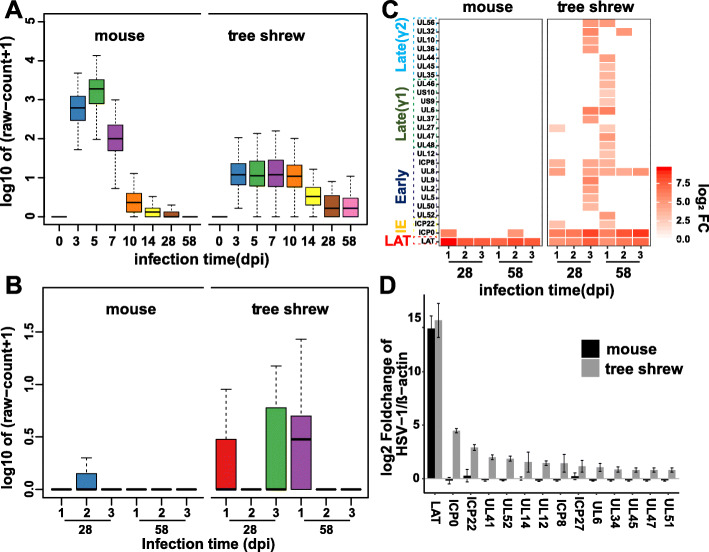
Detection of spontaneous reactivation in animal models. **a** Distribution of read counts for all viral genes in each time point. Viral genes with the mean raw count of three biological replicates value > 0 were selected for mapping. Each individual plot represents the value of log10 (mean + 1). **b** Distribution of read counts for all viral genes in each latent sample (28dpi and 58dpi), and parameter settings as described in (**a**). **c** The heat map shows viral genes that are significantly transcribed (log_2_ fold change > 2) in each latent sample. The graphical layout is the same as in Fig. 2a. **d** RT-qPCR was used to validate the viral genes in the reactivated samples. Error bars denote SD of two biological replicates. 28dpi 1# & 58dpi 2# in the mouse samples, and 28dpi 3# & 58dpi 1# in the tree shrew samples were selected for this experiment

RNA sequencing data suggested that low levels of viral transcripts were widely present in infected tree shrew samples as could be seen in samples beyond day 14, contrasting that of mouse TGs, where the average reads are close to zero (Fig. 4a). To understand the distribution of low levels of viral genes in tree shrew TGs, we separated the three replicates of latency samples and analyzed them individually. We found that the low levels of viral transcripts were from individual tree shrew TGs and not a result of uniform low level of expression in all samples. As seen in Fig. 4b, sample #3 from day 28 and #1 from day 58 appear to express many more viral genes than the remaining samples.

We then calculated the significantly transcribed viral genes in these sample using an R package, DEGseq, which allowed us to identify differentially expressed genes in each latent sample individually [27]. Several viral genes, such as ICP8, UL6, UL8 were found to be differentially expressed in more than one latent tree shrew samples (Fig. 4c), and therefore we validated some selected viral genes using RT-qPCR from 58dpi samples (Fig. 4d). The results indicate that tree shrew #3 from day 28 sample and #1 from day 58 sample are likely undergoing spontaneously reactivation as predicted from our earlier study analyzing tree shrew eye swabbing [5].

### Human and tree shrew are similar in HSV-1 ICP0 and LAT expression pattern in latently infected TGs

Studies using the mouse and other models have established that during HSV-1 latent infection, almost all viral genes are silenced with the exception of the LAT region, which is expressed and produces a stable intron [28, 29], small and microRNAs [8, 30], and helps to maintain latency or reactivation. This is indeed the case in mouse with HSV-1 transcriptome profiling over the course of 2 months post infection. However, viral gene expression in infected tree shrew TGs, with the additional expression of ICP0 transcript during latency, differs significantly from that in mouse TGs. This is evident in Fig. 2a, where both the LAT and the ICP0 region were transcribed at robust levels in tree shrews during latency, whereas only LAT signal was found in mouse.

**Fig. 5:**
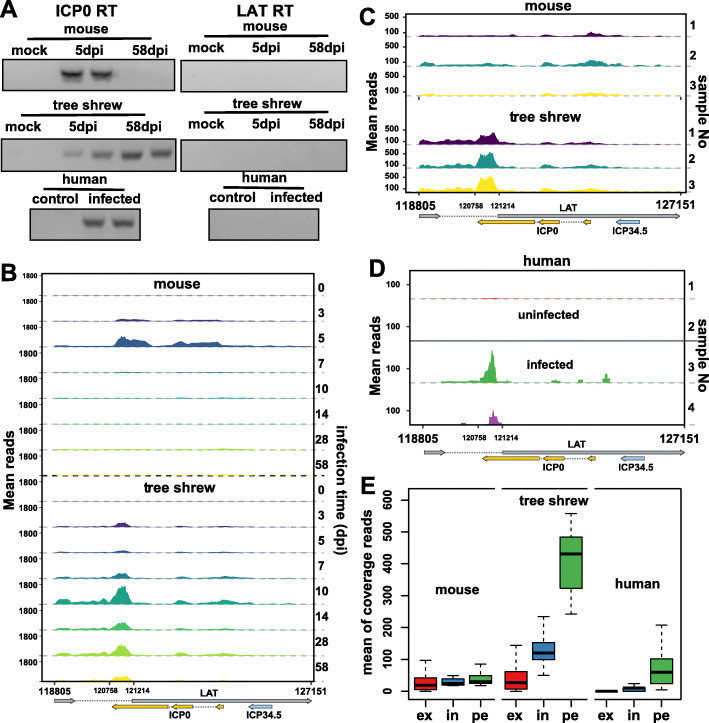
Comparison of LAT transcription patterns during latency between human and animal models. **a** Gel electrophoresis shows that an ICP0 fragment can be amplified in latent tree shrew and human samples, but not latent mouse samples. A strand specific primer was used for ICP0 reverse transcription. The primer sequence of RT primers and PCR primers were both listed in supplemental Table 2. **b** Coverage profiles for the LAT region at indicated days post-infection are shown. The mean reads were calculate using three biological replicates. **c** Showing the read coverage for LAT region of each 58dpi sample respectively and **d** for the human samples we collected (1#&2# uninfected samples; 3#&4# infected samples). **e** Box plots displaying the coverage distribution at three locations within LAT region. ex: LAT exon; in: LAT intron (remove peak region); pe: peak region (120,758-121,214 bp)

To determine what HSV-1 genes are expressed in human TGs and which model is more similar to human in terms of ICP0 and LAT expression patterns during latency, we analyzed human TG sequencing data, and found that the LAT region and ICP0 region were also activated in human TGs, and no other viral genes were detected. To confirm that the observed ICP0 transcription was not an artifact of the specific analytic tool, and also to account for the fact that the region of the genome encoding ICP0 overlaps the LAT region, we used individual strand specific primers for ICP0 and LAT and validated the expression of ICP0 (Fig. 5a), and the strand specificity of this approach had been demonstrated in supplemental Figure 4. When this assay was applied to amplify a longer ICP0 transcript (657pb), we only detected the ICP0 signal in the latently infected tree shrew samples (supplemental Figure 4C). In our previous study, ICP0 transcript was also detected by in situ hybridization in 58dpi tree shrew TGs [5]. Human TGs were harvested a considerable time (1.5–2.5 days) after death, which may result in HSV-1 reactivation, but no viral genes other than LAT and ICP0 were detected in the RNAseq data, and whether this data can really reflect the latent state in the living human is a question we cannot answer.

Although most viral genes were at lower levels in tree shrew than that in mouse during acute stage, the level of LAT was higher in tree shrew TGs than in mouse TGs. This trend continued into latency where LAT is expressed at a much higher level in tree shrew TGs than in mouse (Fig. 5b, c). To reveal the difference of viral gene expression during latency, we compared the LAT region transcription patterns between human and animal models. Two of four human samples we collected (Fig. 5d) and 2 of 16 GEO data (Supplemental Figure 5) provided significant LAT signals. Moreover, the LAT transcription patterns were more similar to those of tree shrews, and they both had a transcription peak in the 120,758–121,214 region, which was not observed in the mouse latency samples (Fig. 5c, d). We also compared the coverage of the peak region (120,758-121,214), LAT intron (remove peak region) and LAT exon (Fig. 5e), and tree shrew and human have higher readings in the peak region. Considering that the animal model is artificially infected, and the human sample is a natural infection, which may result in a low LAT reading in human samples.

## Discussion

HSV-1 infection in tree shrew TGs differs from that in mouse, and viral transcriptional level in tree shrews and mice are affected by many factors. In our previous relative HSV-1 genome quantitative data, acutely infected mouse TGs contained higher levels of viral genome than tree shrew TGs [5], which also contribute to the differences of viral transcription. Another factor that cannot be ignored is whether HSV-1 replicates effectively in the eye of the tree shrew model, because surface replication is the source of the virus transported into the ganglion. In fact, we had tested HSV-1 titer and genome copy number in tree shrew eyes within a period of 46 days after ocular infection (Li et al., in press), and these results support that HSV-1 replication in tree shrew eyes could be comparable with that in mouse eyes.

An important finding from this study is the apparent spontaneous reactivation from latency from two of the tree shrew TGs sequenced, a result supporting our previous study where live virus could be recovered from eye tears in latently infected animals---a feature that mimics human infections seen in tree shrew but less efficient in mouse. From genes detected during spontaneous reactivation, we found immediate early genes ICP0, ICP22 and ICP8, which are important for viral early transcription and genome replication. However, a number of studies support the spontaneous reactivation of infectious HSV-1 in murine sensory ganglia [31, 32], and ICP0 signal was detected in individual latently infected mice in our RNAseq data, but other lytic viral genes reads are too low to tell if they are positive.

What caused the virus to spontaneously reactivate is currently unknown. LAT is the only known transcript in latently infected tissues [10, 33, 34], but it is not just a latency marker, it maintains viral latency [35, 36] by inhibiting apoptosis [37–40], repressing lytic gene expression [41], etc. There have been many studies supporting LAT is essential for the highly induced reactivation phenotype in the mouse model [42–44], and for the high induced or spontaneous reactivation in the rabbit model [45–53]. If true, then the high level of LAT transcript in tree shrew TGs compared to mouse could partially explain why the virus spontaneously reactivates in tree shrew but not in mouse.

In our analyses comparing latently infected mouse, tree shrew and human TGs, we observed that LAT could be detected from all three, but tree shrew TG was more similar to human TG in that both have high reads in a LAT intron region. Considering that the oligo (dT) method cannot enrich non-polyadenylated LATs, the high level of LAT signal are mainly supported by polyadenylated LATs [54–57], the signal in the LAT intron region is in fact ICP0, but not LAT intron, which is not polyadenylated. This is supported by the strand-specific RT-qPCR experiments shown in Fig. 5a.

A more important similarity between infected tree shrew and human TGs is that both express transcript from the ICP0 region. In contrast, there are no ICP0 transcripts detected in infected mouse TGs, which is inconsistent with some previous reports that the ICP0 signal they detected was located near the first exon at the 5′ end [58, 59]. In light of this, this comparison could also include infected rabbits TGs in future analysis to examine whether this ICP0 transcripts exist in reactivating samples.

Both in tree shrew and human TGs, most of the ICP0 reads are concentrated at the 3′ end of the ICP0 gene (Fig. 5c & d), while the remaining region of ICP0 has very few reads, raising the possibility that this region is independently transcribed. However, it is also possible that the uneven reads were a result of the high GC content of the HSV genome. Since we could not amplify the full-length ICP0 transcript, we could not rule out the possibility of partial transcripts arising from the 3′ of ICP0. In addition, there is a 0.8 kb LAT insulator and a CTCF binding site within LAT intron region [60], and these elements are both located up-stream of the LAT/ICP0 transcription peak, making it possible that the CTCF binding site could serve as a promoter to for this transcript, as reporter by others [61].

## Conclusion

In this study, we compared the viral transcriptome of infected mouse and tree shrew TGs during the course of infection, and we compared the pattern of viral transcripts in latently infected mouse and tree shrew TGs with that of human TGs. We found that HSV-1 transcription in acutely infected TGs differs dramatically between mouse and tree shrew, with HSV-1 in mouse TG undergoing productive infection, while that in tree shrew TGs appears to be going through an abortive infectious cycle, missing keys genes needed for viral transcription, replication and maturation. During the latent phase of infection, LAT was detected in mouse, tree shrew and human TGs, but we also detected an ICP0 transcript fragment from tree shrew and human TGs, making tree shrew latent infection more similar to human than mouse. When we analyzed tree shrew TGs individually, we found samples that appeared to be undergoing spontaneous reactivation. Together these analyses support the tree shrew as a better model of human HSV-1 infection in the peripheral nervous system, offering the possibility of a better understand HSV-1 latency and reactivation, and the discovery of potential novel targets for therapeutic interventions. Taken together, the transcriptome data reveals that tree shrews and humans have a more similar transcription pattern in the LAT region during latency than that of mice and humans, supporting tree shrews as a more accurate animal model for research on HSV-1 latency and reactivation.

## Materials and methods

### Experimental animals

Chinese tree shrews, 6-month-old female, were obtained from the experimental animal core facility of the Kunming Institute of Zoology, Chinese Academy of Sciences. During the experiment, tree shrews were kept in experimental cages of 54 cm × 45 cm × 50 cm, and no more than two animals per cage. The temperature in the room was controlled at 15 ~ 28 °C, the relative humidity was 40 ~ 70%, the daily light was 12 h, and the noise should not exceed 60 dB. BALB/C mice, 6-week-old female, were obtained from the Kunming Medical University. All experimental procedures and animal care were carried out in accordance with the protocols approved by the Institutional Animal Care and Use Committee of the Kunming Institute of Zoology, Chinese Academy of Sciences. The research program was reviewed and approved by the Institutional Animal Care and Use Committee of Kunming Institute of Zoology, Chinese Academy of Sciences.

### Virus and cells

HSV-1 strain 17+ was used to infect mouse and tree shrews. Viral culture was performed in Vero cells and titrated by plaque forming assay on RS1 cells, and these two cells were both obtained from Conservation Genetics Academy of Science (CAS) Kunming Cell Bank. The infected cells were cultured in DMEM supplemented with 2% fetal bovine serum (GibcoTM). All cells were maintained at 37 °C with 5% CO2. Experiments involving infectious virus were conducted in a Biosafety level 2 laboratory.

### Animal infections and collection of samples

Animal anesthesia, corneal scarification and inoculation with HSV-1 17+ virus was as previously described [5]. 1 × 10^4^ PFU of HSV-1 were used to inoculate each mouse eye, and 1 × 10^6^ PFU were used on tree shrews, which was consistent with our previous reports [5, 19]. The control (mock infected) animals were also scratched and treated with Vero cell supernatant. To generate biological replicates, three groups of animals were infected independently on different days. Samples of infected trigeminal ganglions were collected at 3, 5, 7, 10, 14, 28, 58dpi, and mock infected samples were collected after 24 h of treatment, and then were ground to a fine powder in liquid nitrogen using 50 ml grinding beakers and 20-mm grinding ball for RNA extraction.

### Human trigeminal ganglions collection and preparation

Four human trigeminal ganglia were obtained at autopsy and provided by Academy of Forensic Science (Shanghai, China) and Second Affiliated Hospital of Kunming Medical University. According to the record, the material was taken after 2–3 days of death, then stored in liquid nitrogen and transported with dry ice, and demographics are shown in supplemental Table 3. This is about as early as it is possible to legally obtain human tissue for our studies. Tissues were stored at − 80 °C until processing. To disrupt the tissue, frozen ganglia were wrapped in aluminum foil and mechanically broken apart on dry ice using a metal anvil and hammer, both also frozen on dry ice. Small fragments of tissue from a trigeminal ganglion from were used for RNA extraction.

### RNA extraction and sequencing

Mouse, tree shrew or human TGs were individually ground to a fine powder in liquid nitrogen before RNA extraction. For each sample, approximately 1 g of powder (from two TGs of one animal) was resuspended in 1 ml TRIzol reagent (Life Technologies) and total RNA was extracted according to the manufacturer’s recommendation. Afterwards, RNA samples were sent to BGI (Wuhan, China) for purification, library preparation and sequencing. The Ambion Trubo DNA-free kit (Life Technologies) was used to eliminate genomic DNA contamination, and an Agilent 2100 Bioanalyzer (Agilent RNA 6000 Nano Kit) to perform the total RNA sample quality control. mRNAs were isolated from total RNA using the oligo (dT) method, and then purified and fragmented using divalent cations under elevated temperature. The oligo (dT) method could not extract, and thus excluded non-polyadenylated transcripts, such as non-polyadenylated LAT introns, and retains only polyadenylated LATs [54–57]. First strand cDNA was synthesized using random primers, and the second strand cDNA was synthesised with Polymerase I and RNase H. cDNA fragments were purified and resolved with EB buffer for end reparation and single nucleotide A (adenine) addition. After that, the cDNA fragments were linked with adapters. Those cDNA fragments with suitable size (300 bp) were selected for PCR amplification. An Agilent 2100 Bioanaylzer and ABI StepOnePlus Real-Time PCR System were used in quantification and qualification of the libraries. Finally, all of the transcriptome libraries were sequenced using an Illumina HiSeq X Ten sequencer with a paired-end protocol.

### Read mapping, normalization, and statistical analysis of differential gene expression

According to the BGI’s instructions, the low quality reads (More than 20% of the bases qualities were lower than 10), reads with adaptors and reads with unknown bases (N bases more than 5%) were filtered using an internal software, SOAPnuke, to get the clean reads. After filtering, the remaining reads were stored in FASTQ format. All reads were aligned to the host genome or HSV-1 17+ using HISAT2 software with default parameters [62]. In viral transcriptome analysis, all reads aligned to host genomes were filtered out, and all unmapped reads were then mapped against HSV-1 17+ genome. The quantification of transcript abundance (raw count) was conducted using featureCounts [63] software supported by the Subread package [64], and multi-mapping and multi-overlapping reads were excluded from expression analysis. We chose to normalize our data set using the relative log expression method implemented in the DESeq2 package (v1.22.2) [20] in R (www.r-project.org), and only viral genes that had a read count of at least 5 were considered. After DESeq2 analyses, genes with a log2 fold change > 1 and FDR- adjusted p-value <− 0.01 were considered differentially expressed.

### Reverse transcription PCR (RT-PCR) and quantitative real-time RT-PCR (RT-qPCR)

In Fig. 5a and b, reverse transcription used strand-specific primers for ICP0 and LAT transcript respectively and was performed with a high fidelity RT-PCR kit (TaKaRa), according to the manufacturer’s instructions. In a 20 μl reaction mixtures contained RNase inhibitor, RTase, PrimeScript buffer, dNTP mixture, 1 μg template RNA and 5pM a strand-specific RT primer. After RT reaction, 1 μL cDNA was used for PCR reaction flowing by 30 cycles of 10 s at 98 °C, then 5 s at 60 °C, and 20s at 72 °C. The RT-PCR product was electrophoresed on a 2% agarose gel for 20 min at 200 V. For gene expression analysis via RT-qPCR, isolated total RNA was reverse-transcribed using a PrimeScriptTM RT reagent Kit with gDNA Eraser (TaKaRa). The RT-qPCR was performed in an Applied Biosystems 7900HT using FastStart Universal SYBR Green Master (ROX, Roche). Per RT-qPCR reaction, cDNA derived from 50 ng RNA was deployed. Cycling conditions were 10 min 95 °C, followed by 45 cycles of 10s at 95 °C, 30s at 60 °C, and 30s at 72 °C. Primers are listed in supplemental Table 2. For the expression of fold changes of RT-qPCR data, the 2 − ΔΔCt method was used [65].

## Supplementary information

**Additional file 1.** Supplementary figures.

## Data Availability

The HSV-1 reference genome and annotation file were downloaded from NCBI (ftp://ftp.ncbi.nlm.nih.gov/genomes/all/GCF/000/859/985/GCF_000859985.2_ViralProj15217), GCF_000859985.2_ViralProj15217_genomic.fna.gz and GCF_000859985.2_ViralProj15217_genomic.gff.gz). The RNA-seq data generated for this paper have been submitted to the Genome Sequence Archive [66] in BIG Data Center [67], Beijing Institute of Genomics (BIG), Chinese Academy of Sciences (GSA, http://bigd.big.ac.cn/gsa), under accession number CRA001750. The datasets used and analysed during the current study are available from the corresponding author on reasonable request.

## Abbreviations

HSV: Herpes simplex virus
TG: Trigeminal ganglia
HSV-1: Herpes simplex virus-1
dpi: days post infection
LAT: Latency-associated transcript
CAS: Conservation Genetics Academy of Science
RT-PCR: Reverse transcription PCR
RT-qPCR: Quantitative real-time RT-PCR
DEG: Differentially expressed gene
