# Correction to: Longitudinal transcriptomic characterization of viral genes in HSV-1 infected tree shrew trigeminal ganglia

**DOI:** 10.1186/s12985-020-01419-6

**Published:** 2020-10-08

**Authors:** Erlin Wang, Yunshuang Ye, Ke Zhang, Jinlong Yang, Daohua Gong, Jianhua Zhang, Shijun Hong, Huan Zhang, Lihong Li, Guijun Chen, Liping Yang, Jianmei Liu, Hanyu Cao, Ting Du, Nigel W. Fraser, Le Cheng, Xia Cao, Jumin Zhou

**Affiliations:** 1 Key Laboratory of Animal Models and Human Disease Mechanism of the Chinese Academy of Science/Key Laboratory of Healthy Aging Research of Yunnan Province, Kunming Institute of Zoology, the Chinese Academy of Sciences, Kunming, 650223 Yunnan China; 2 Key Laboratory of Second Affiliated Hospital of Kunming Medical University, Kunming, 650000 Yunnan China; 3 BGI-Yunnan, BGI-Shenzhen, Kunming, 650000 Yunnan China; 4 Kunming College of Life Science, University of Chinese Academy of Sciences, Beijing, 100049 China; 5 Shanghai Key Laboratory of Forensic Medicine, Shanghai Forensic Service Platform, Academy of Forensic Science, Shanghai, 200063 China; 6 School of Forensic Medicine, Kunming Medical University, Kunming, 650101 Yunnan China; 7 Department of Microbiology, Perelman School of Medicine, University of Pennsylvania, Philadelphia, PA 19104 USA; 8 Department of Medicine Laboratory, Fuwai Central China Cardiovascular Hospital, Zhengzhou, 450003 Henan China; 9 College of Forensic Science, Xi’an Jiaotong University, Xi’an, 710049 Shaanxi China

## Correction to: Virology Journal (2020) 17:95 https://doi.org/10.1186/s12985-020-01344-8

Following publication of the original article [1], the authors identified an error in the author name of Shijun Hong. Also, would like to correct the Funding section. In this Correction, correct and incorrect versions are shown.

The incorrect author name is: Renjun Hong.

The correct author name is: Shijun Hong.

The author group has been updated above.

**Funding**

The sentence currently reads:

This study was supported by grants from National Natural Science Foundation of China-Yunnan Joint Found (NSFC, U1602226), National Natural Science Foundation of China (NSFC, 81672040) and Ministry of Science and Technology of China (MOST, SQ2018YFE020419) and a Thousand Foreign Talent scholarship from Yunnan province to Jumin Zhou, Yunnan applied basic research project (2017FE467) and International Science and Technology Cooperation Project (2017IB011) to Xia Cao and Chinese Academy of Sciences President’s International Fellowship Initiative (PIFI, 2019VBA0045) to Nigel W. Fraser.

The sentence should read:

This study was supported by grants from National Natural Science Foundation of China-Yunnan Joint Found (NSFC, U1602226), National Natural Science Foundation of China (NSFC, 81672040), Ministry of Science and Technology of China (MOST, 2018YFC2000400, 2018YFE0203700), CAS “Light of West China” Program (xbzg-zdsys-201909) and a Thousand Foreign Talent scholarship from Yunnan province to Jumin Zhou, Yunnan applied basic research project (2017FE467) and International Science and Technology Cooperation Project (2017IB011) to Xia Cao, National Science Foundation for Young Scientists of China (NSFC, 31802026) to Lihong Li and Chinese Academy of Sciences President’s International Fellowship Initiative (PIFI, 2019VBA0045) to Nigel W. Fraser.
