# Supplementary material for: Longitudinal transcriptomic characterization of viral genes in HSV-1 infected tree shrew trigeminal ganglia
Source: Virol J. 2020 Jul 8;17:95. doi: 10.1186/s12985-020-01344-8 (PMC7341572; doi:10.1186/s12985-020-01344-8)
Supplement: Supplementary file 1 — Additional file 1. Supplementary figures. [file 12985_2020_1344_MOESM1_ESM.docx]

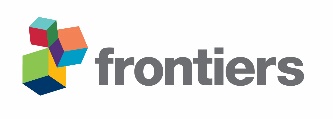
 Supplementary Material


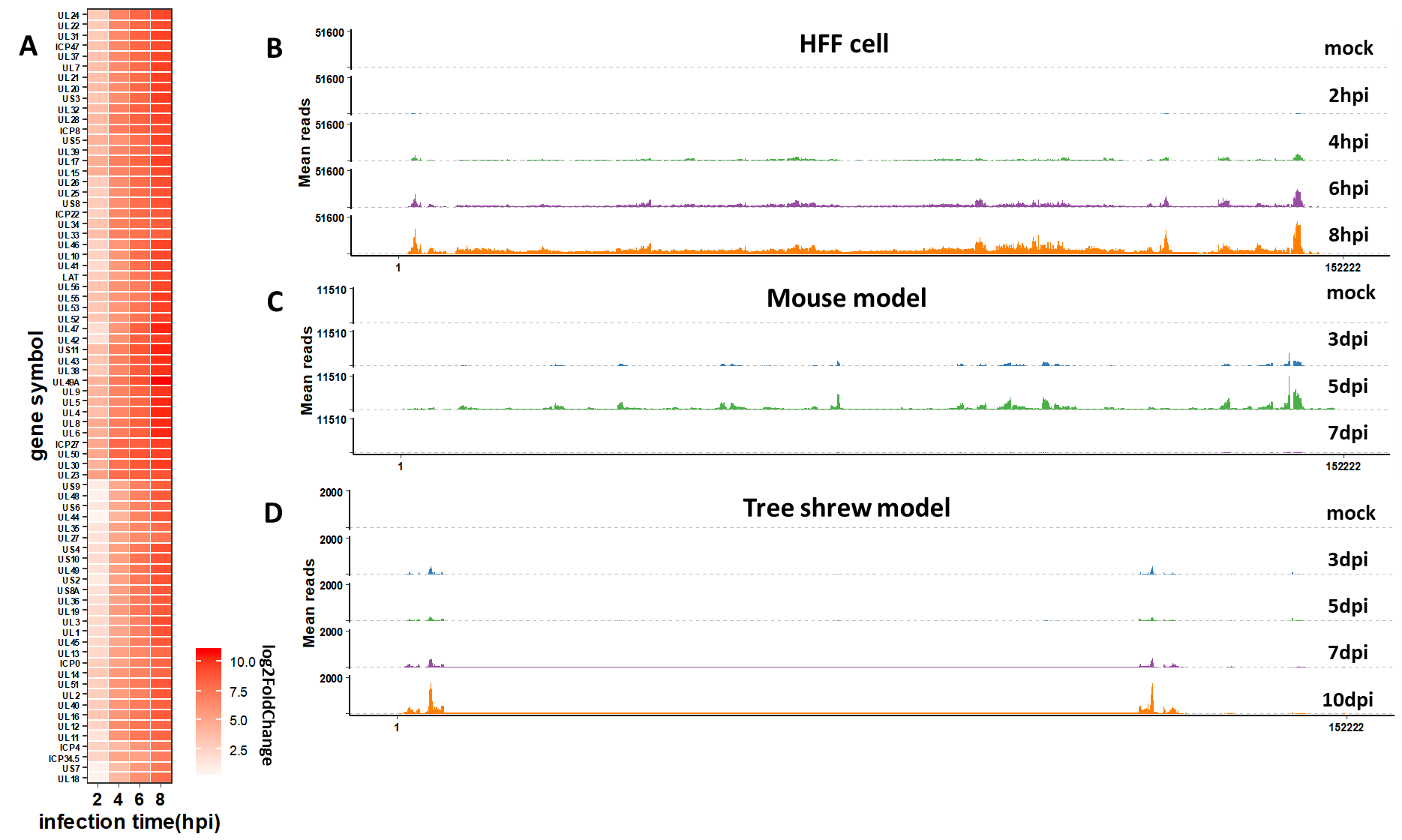


**Figure S1-figure supplement. HSV-1 transcriptional profiler in HFF cell.**

**A)** HFF cells were infected with WT HSV-1 for 2h, 4h, 6h and 8h. RNA-Seq data as log2 fold change and heatmap of viral genes that are differently expressed between HSV-1 infection and mock infection. Displayed DEGs had log2 fold change > 1.5 and FDR-adjusted p value < 0.01. **B)** HSV-1 viral genome coverage detected by RNA-seq in in-vitro-infected HFF cells at indicated hours post-infection (average of 2 replicates). RefSeq gene annotation is indicated below (red represents LAT region). Arrowed boxes indicate transcription directions and coding regions. The transcribed strand is indicated by the direction of the arrowheads. RNA-seq dataset [1] of HSV-1 infected HFF cells with MOI =10 were downloaded from the NCBI Gene Expression Omnibus (GEO) database with accession number GSE59717, which included 2 replicates for each experiment. Alignment was performed using bedtools software. RNA-seq read coverage (average of 3 replicates) for HSV-1 genome in the mouse **(C)** and tree shrews **(D)** model.


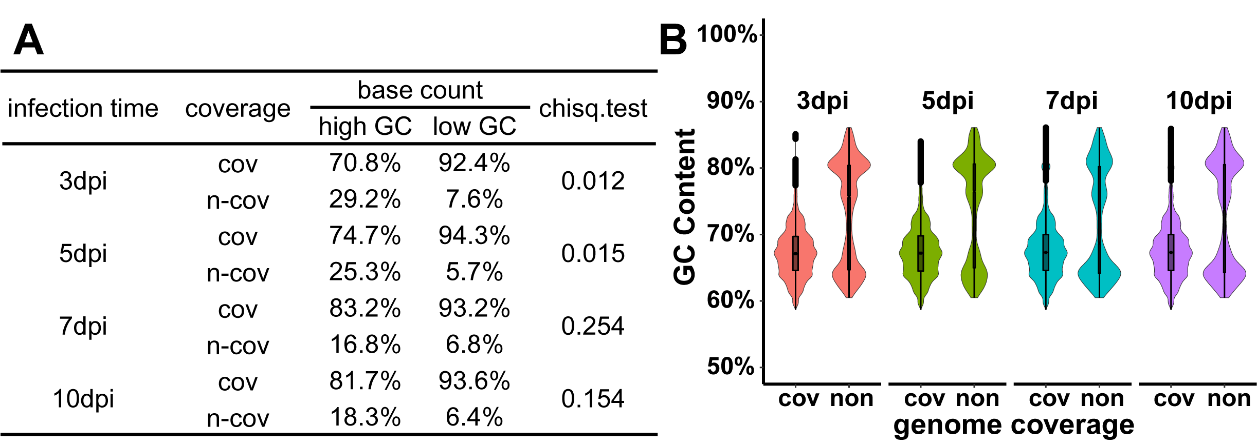


**Figure S2-figure supplement. Analysis of the effect of GC content on reads coverage in whole HSV-1 genome.**

**A)** This experimental parameter settings are consistent with the figure 3D, and the analysis region is extended to whole HSV-1 genome. **B)** Violin plot showing the distribution of GC content and reads coverage statue in whole HSV-1 genome region. Labels are the same as in figure 3E.

**
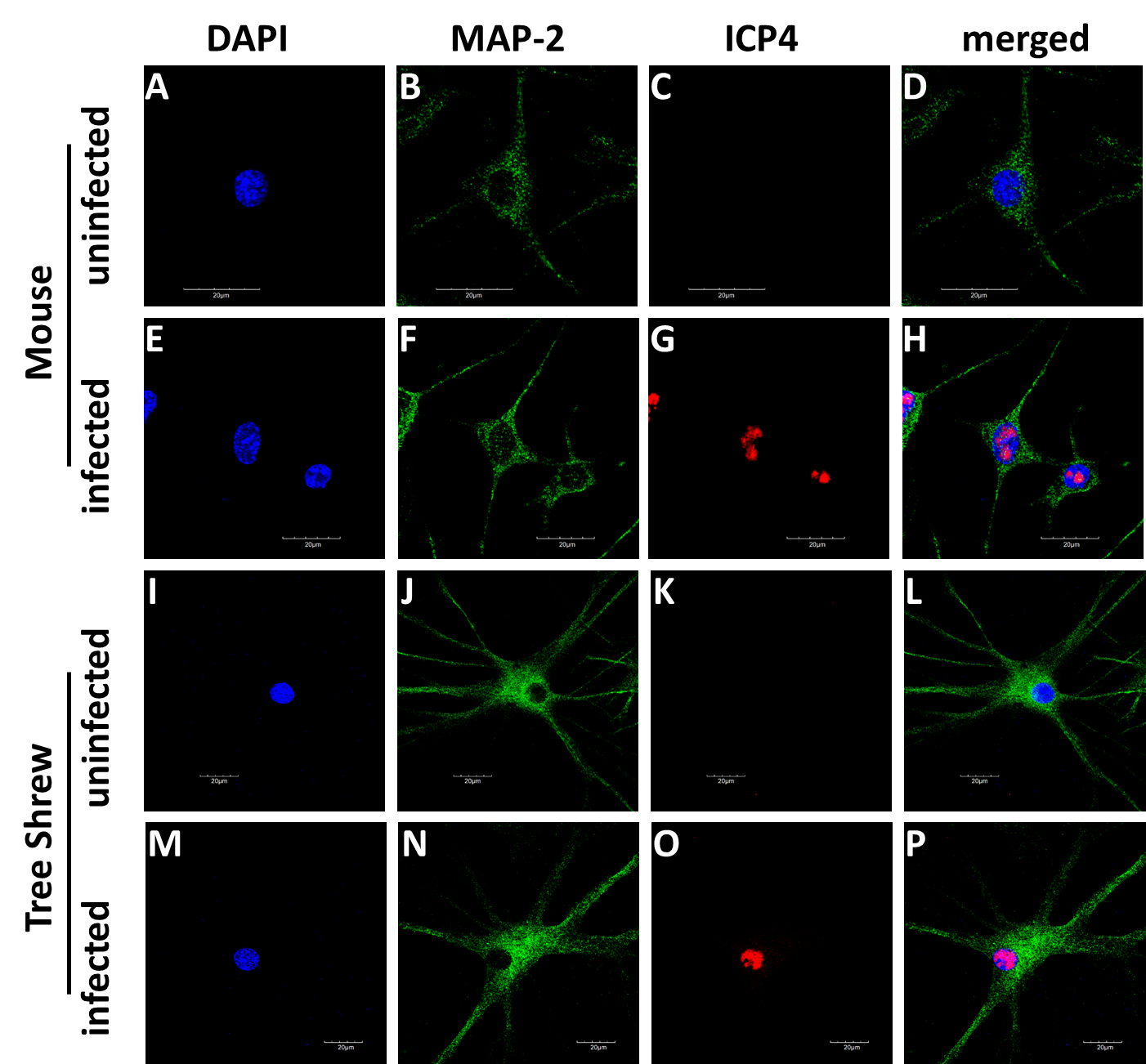
F****igure S3-figure supplement. HSV-1 infects primary trigeminal ganglion cells in vitro.**

Immunofluorescence staining of ICP4 (red) and MAP2 (green) in mock-infected **(A-D, I-L)** or HSV-1 17+ infected **(E-H, M-P)** primary neuron isolated from mouse **(A-H)** and tree shrews (I-P) trigeminal ganglion (TG) respectively at 6 hours post infection. ICP4 positive cells shows successful HSV-1 infection, and ICP4 negative cells shows no infection.

**Figure S4-figure supplement. RT-PCR to test the transcription of LAT and ICP0**

**A)** Schematic view of approach for strand-specific reverse transcription and reaction-specific validation. **B)** Primers used in the RT-PCR detection of viral transcripts. Strand-specific reverse transcription was performed with primer LAT RT or ICP0 RT alone. The cDNAs were then PCR amplified with the addition of primer “LAT testing” or “ICP0 testing” respectively. **C)** Amplification results of strand-specific reverse transcription cDNAs. In the acute phase, 5dpi mouse and tree shrew TG total RNA was used for reverse transcription, and in the latent phase, 58dpi mouse and tree shrew samples. Two infected human samples were also used. For the LAT cDNA, LAT RT + LAT testing primers were used. At least two repeated experiments were done, and the figure shows one of the results. For the ICP0 cDNA, ICP0 RT + ICP0 testing primers were used. L: LAT cDNA, I: ICP0 cDNA.


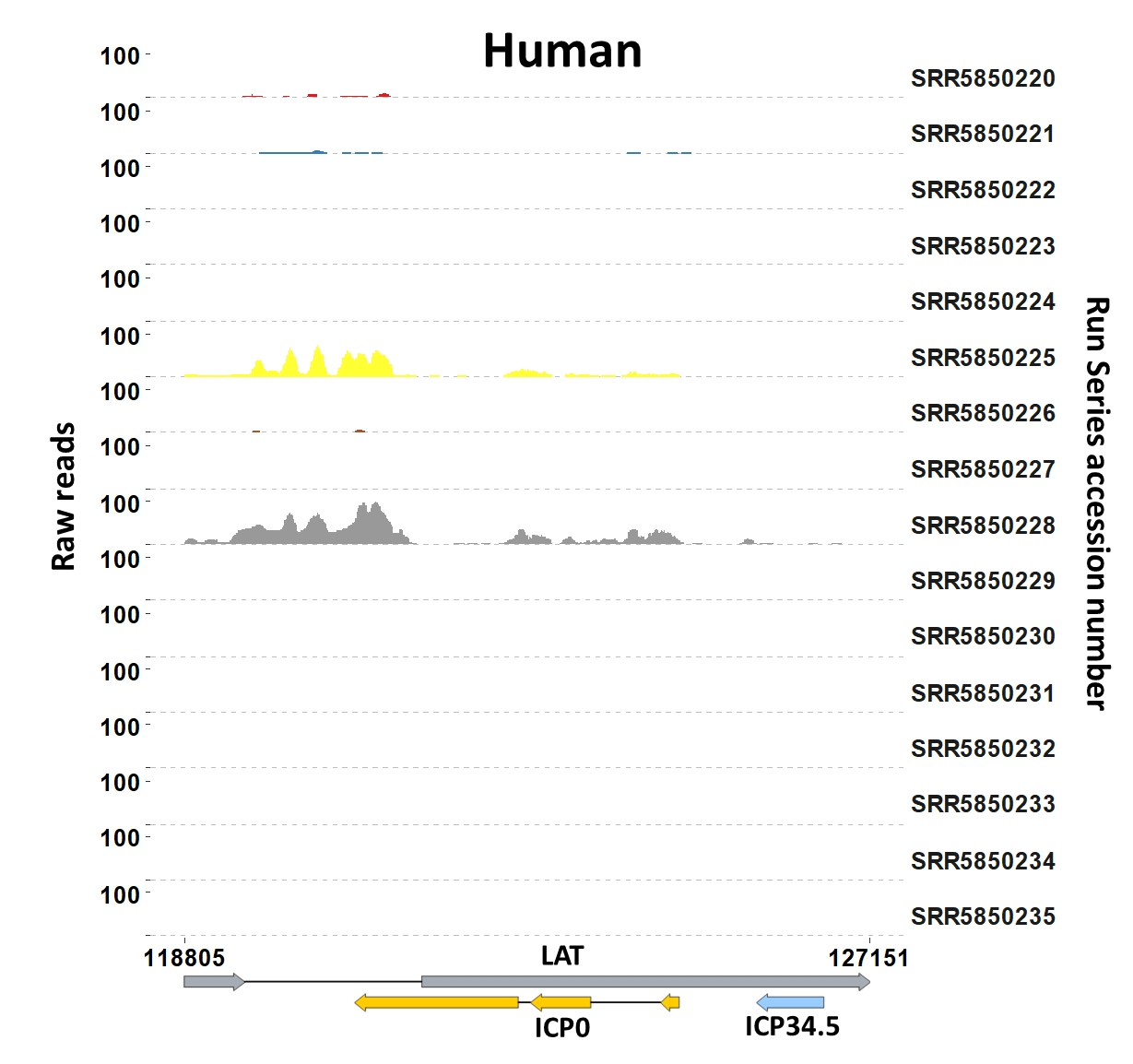


**Figure S5-figure supplement. HSV-1 LAT transcriptional profiler in human TGs.**

Read coverage for the LAT region in 16 human TGs, and each row represents a sample. The max reads count was indicated on the y-axis. LAT region annotation is indicated below. These human TGs RNA-seq data have been published in NCBI database (Accession: PRJNA384203) [2].

**SUPPLEMENTARY TABLES**

**Table S1. HSV-1 genes function description from NCBI and UniPort annotation.**

| **Symbol** | **Phase** | **Product** | **Function** |
| --- | --- | --- | --- |
| **US3** | Late(γ1) | serine/threonine protein kinase US3 | tegument protein; phosphorylates nuclear egress lamina protein; mediates phosphorylation of HDAC1 and HDAC2 and other cellular and viral proteins; involved in protein phosphorylation; involved in apoptosis; involved in nuclear egress |
| **UL11** | Late | myristylated tegument protein | envelope-associated; involved in virion morphogenesis |
| **UL13** | Late | tegument serine/threonine protein kinase | involved in protein phosphorylation |
| **UL14** | Late(γ2) | tegument protein UL14 | involved in virion morphogenesis |
| **UL21** | Late | tegument protein UL21 | interacts with microtubules; involved in virion morphogenesis |
| **UL41** | Late(γ1) | tegument host shutoff protein | mRNA-specific RNase; involved in cellular mRNA degradation |
| **US2** | Late(γ2) | virion protein US2 | possibly envelope-associated; interacts with cytokeratin 18 |
| **US5** | Late | envelope glycoprotein J | type 1 membrane protein; contains a signal peptide |
| **UL20** | Late(γ1) | envelope protein UL20 | type 3 membrane protein; 4 transmembrane domains; involved in virion morphogenesis; involved in membrane fusion |
| **UL43** | Late | envelope protein UL43 | type 3 membrane protein; possibly involved in membrane fusion;11 transmembrane domains |
| **UL49A** | Late(γ2) | envelope glycoprotein N | type 1 membrane protein; contains a signal peptide; complexed with envelope glycoprotein M; involved in virion morphogenesis; involved in membrane fusion |
| **UL53** | Late(γ1) | envelope glycoprotein K | type 3 membrane protein; contains a signal peptide; 4 transmembrane domains; involved in virion morphogenesis; involved in membrane fusion |
| **UL15** | Late | DNA packaging terminase subunit 1 | contains an ATPase domain; involved in DNA encapsidation;transiently associated with maturing capsids |
| **UL28** | Late | DNA packaging terminase subunit 2 | involved in DNA encapsidation;transiently associated with maturing capsids |
| **UL33** | Late(γ2) | DNA packaging protein UL33 | interacts with DNA packaging terminase subunit 2; involved in DNA encapsidation |
| **UL32** | Late(γ2) | DNA packaging protein UL32 | involved in DNA encapsidation; possibly involved in capsid transport |
| **UL17** | Late | DNA packaging tegument protein UL17 | capsid-associated; involved in DNA encapsidation; involved in capsid transport |
| **UL24** | Late(γ1) | nuclear protein UL24 | may participate in nuclear egress of viral particles. |
| **UL4** | Late(γ2) | nuclear protein UL4 | colocalizes with regulatory protein ICP22 and nuclear protein UL3 in small, dense nuclear bodies |
| **UL55** | Late(γ2) | nuclear protein UL55 | none |
| **ICP4** | Immediate Early | transcriptional regulator ICP4 | involved in gene regulation |
| **UL9** | Early | DNA replication origin-binding helicase | involved in DNA replication |

**Table S2. List of qRT-PCR primers.**

| **Target** | **primer sequence** | | **Accession No. (Nucleotide No.)** |
| --- | --- | --- | --- |
| **UL21** | **Fwd:** | CAGCTCCTCATGTTCGACCT | NC_001806.2 (43302-43409) |
|  | **Rev:** | GGCGAGACGTTGCGATTC |  |
| **UL17** | **Fwd:** | CGACTCCGACGGCCATTA | NC_001806.2 (31630-31756) |
|  | **Rev:** | GCGACATTTACTCTGCTCGC |  |
| **ICP4** | **Fwd:** | CTGATCACGCGGCTGCTGTACA | NC_001806.2 (148188-148332) |
|  | **Rev:** | CGGTGATGAAGGAGCTGCTGTTGC |  |
| **UL4** | **Fwd:** | GTACGCGTATCCGTTCGACT | NC_001806.2 (12044-12133) |
|  | **Rev:** | GTCCAGACCACGGTCAAAAT |  |
| **UL43** | **Fwd:** | GTCAAGACGGTGCGAGAACA | NC_001806.2 (95636-95822) |
|  | **Rev:** | CCAACCCGAGGTTGATGACC |  |
| **UL55** | **Fwd:** | TTAGCGTCGGGACGATTAAG | NC_001806.2 (115834-115938) |
|  | **Rev:** | AGAACGGATACCGACAGTGG |  |
| **ICP22** | **Fwd:** | GACCCCCGACCTGGGCTACAT | NC_001806.2 (133207-133503) |
|  | **Rev:** | GGGCCGTACCGTCTGGTCTCC |  |
| **UL41** | **Fwd:** | GGACATCCGCGACGAAAAC | NC_001806.2 (91240-91317) |
|  | **Rev:** | AGAAACCTGTCGGCGATATCAG |  |
| **UL52** | **Fwd:** | GACCCCGCTACAACCGGACCAC | NC_001806.2 (109450-109625) |
|  | **Rev:** | CCACGAGCGAGCGTAGGGAC |  |
| **UL12** | **Fwd:** | AGCTCCGGGGATCTGGTC | NC_001806.2 (25302-25448) |
|  | **Rev:** | CCTGCCGATAAACGTCACCA |  |
| **UL14** | **Fwd:** | GACCAACGCTTCTCTGTGGG | NC_001806.2 (28405-28545) |
|  | **Rev:** | GGATGCGAGCCAATCCTTGA |  |
| **ICP8** | **Fwd:** | CGACAGTAACGCCAGAAG | NC_001806.2 (61941-62052) |
|  | **Rev:** | GGAGACAAAGCCCAAGAC |  |
| **ICP27** | **Fwd:** | ATGTGCATCCACCACAACCT | NC_001806.2 (114827-115005) |
|  | **Rev:** | TCCTTAATGTCCGCCAGACG |  |
| **UL6** | **Fwd:** | TCCTCAACGCCACCACTTAC | NC_001806.2 (15360-15530) |
|  | **Rev:** | TCGTCCTAAGCGTGTTCCG |  |
| **UL34** | **Fwd:** | ACCTGCGCATACAGAACACC | NC_001806.2 (69883-69991) |
|  | **Rev:** | AGGATCACATTGGTCCGCTC |  |
| **UL45** | **Fwd:** | GCATATCCTGGGATCCGACC | NC_001806.2 (98241-98373) |
|  | **Rev:** | GAGGATCTTGCCGACTGGAC |  |
| **UL47** | **Fwd:** | GCTAGAGGCTCTGGAGGAGAT | NC_001806.2 (102858-102974) |
|  | **Rev:** | GCTCGGCGATGGGATGTG |  |
| **UL51** | **Fwd:** | CCCGACGCCTCGTGAAG | NC_001806.2 (108760-108828) |
|  | **Rev:** | GTCGTTCTAGGTTCACCATGCA |  |
| **LAT Intron** | **RT:** | CCCACTACACCAGCCAATCCGTGTC | NC_001806.2 (119998-120150) |
|  | **Fwd:** | GCGCGTGCCTTTGCACACCA |  |
|  | **Rev:** | GAATGCGGTGCAGCCAGAGA |  |
| **ICP0** | **RT:** | GACACGGATTGGCTGGTGTAGTGGG | NC_001806.2 (121387-121455) |
|  | **Fwd:** | CCGCTTCCGCCTCCTC |  |
|  | **Rev:** | GGCCGAGGGAGGTTTCC |  |
| **mmu Actb** | **Fwd:** | CCACTGTCGAGTCGCGTCCA |  |
|  | **Rev:** | GCTTTGCACATGCCGGAGCC |  |
| **tup ACTB** | **Fwd:** | CCTCACAGAGCGCGGCTACA |  |
|  | **Rev:** | CAGCGGAACCGCTCATTGCC |  |

**Table S3. Demographics of the 4 individuals included in this study.**

| **Sample Number** | **Post-mortem interval (h)** | **Manner of Death** | **Gender** | **Age (year)** |
| --- | --- | --- | --- | --- |
| Sample1 | 56 | Accident | M | lost |
| Sample2 | 48 | coronary heart disease | M | 42 |
| Sample3 | 34 | Accident | M | 38 |
| Sample4 | 61 | Accident | M | 32 |

**SUPPLEMENTARY EXPERIMENTAL PROCEDURES**

**Cultivating primary neurons from tree shrews and mouse trigeminal ganglia**

Before commencing the dissection, the 6-well plate was treated with collagen and laminin for 2 hours and then washed twice with ddH2O. The trigeminal ganglion of the mouse or tree shrew was dissected in a sterile environment, placed in D-Hanks buffer and shredded, then the tissue pieces were transferred to a 50 ml centrifuge tube and 4 ml of 4℃ precooled D-Hanks was added. Gently centrifuge the ganglia for 2 min at 1400g and then aspirate the excess media. The tissue was resuspended in serum-free DMEM medium containing 0.25% trypsin. Gently blow, add complete culture medium (DMEM/F12, 1% antibiotics, 1% glutamine, 10%FBS) to neutralize pancreatin after tissue pieces are digested. Centrifuge for 2 min at 1400g to remove trypsin and wash the precipitate twice. Add medium in the tube to resuspend the cells, and then filter the dissociated neurons through a 500 mesh filter into a 50ml conical tube to discard any remaining clumps. Withdraw a 10ul aliquot of the filtered and determination of the number of live cells by trypan blue staining. The cell suspension and 2ml complete culture medium were added to the treated 6-well plate, and waiting in the 37 ℃ incubator for 6 hours, then replacing Neurobasal medium [Life Technologies] for further culturing for 40 hours, and adding B-27 supplement to inhibit glial cells [3].

**Immunofluorescence staining**

Primary neuron were seeded in 24-well plates and allowed to attach and grow for one day. Cell were infected with HSV-1 17+ in MOI=5 and fixed using a 4% paraformaldehyde solution at 6 hours post-infection. Cells were fixed for 15 minutes at room temperature and washed 3 times with PBS, then permeabilized with 0.5% Triton-X solution in PBS for 20 minutes and also washed 3 times with PBS. Cells were then blocked with 10% BSA and incubated with primary antibodies in 5% BSA overnight at 4℃. Cells were washed 3 times each time for 5min, incubated with secondary antibodies in 5% BSA for 1 hour at room temperature, washed three times with PBS and covered with 1ml PBS containing a 1:10,000 dilution of Hoechst 33342 (Life Technologies, cat #H1399). Cell were imaged on a Nikon A1 MP+/A1R MP+ Multiphoton Microscopes. Primary antibodies were mouse monoclonal anti-ICP4 (Self-prepared antibody) and rabbit monoclonal anti-MAP2 (Cell Signaling Technologies, Cat # 4542S, used at 1:100 dilution). Secondary antibodies were Alexa Fluor® 594 Goat Anti-Mouse IgG (H+L) Antibody and Alexa Fluor® 488 Goat Anti-Rabbit IgG (H+L) Antibody (Life Technologies, cat #A11005, #A11034, used at 1:1000 dilution).

**SUPPLEMENTARY REFERENCES**

1. Rutkowski AJ, Erhard F, L'Hernault A, Bonfert T, Schilhabel M, Crump C, Rosenstiel P, Efstathiou S, Zimmer R, Friedel CC, Dolken L: **Widespread disruption of host transcription termination in HSV-1 infection.** *Nat Commun* 2015, **6:**7126.

2. LaPaglia DM, Sapio MR, Burbelo PD, Thierry-Mieg J, Thierry-Mieg D, Raithel SJ, Ramsden CE, Iadarola MJ, Mannes AJ: **RNA-Seq investigations of human post-mortem trigeminal ganglia.** *Cephalalgia* 2018, **38:**912-932.

3. Kobayashi M, Kim J-Y, Camarena V, Roehm P, Chao MV, Wilson AC, Mohr I: **A primary neuron culture system for the study of herpes simplex virus latency and reactivation.** *Journal of visualized experiments: JoVE* 2012.
